# The risk of myelodysplastic syndrome and acute myeloid leukemia by metformin use and type 2 diabetes status – a Danish nation-wide cohort study

**DOI:** 10.2340/1651-226X.2025.42422

**Published:** 2025-05-07

**Authors:** Emelie Curovic Rotbain, Klaus Rostgaard, Katja Kaastrup, Stine Ulrik Mikkelsen, Henrik Hjalgrim, Kirsten Grønbæk

**Affiliations:** a Department of Hematology, Rigshospitalet, Copenhagen, Denmark; b Hematology, Danish Cancer Institute, Danish Cancer Society, Copenhagen, Denmark; c Department of Epidemiology Research, Statens Serum Institut, Copenhagen, Denmark; d Biotech Research and Innovation Centre, University of Copenhagen, Copenhagen, Denmark; e Department of Clinical Medicine, Copenhagen University, Copenhagen, Denmark

**Keywords:** epidemiology, cancer, acute myeloid leukemia, myelodysplastic syndrome, type 2 diabetes, metformin

## Abstract

**Background and purpose:**

The treatment options for myelodysplastic syndromes (MDS) and acute myeloid leukemia (AML) have increased recently. However, drug resistance persists and patients who are ineligible for curative treatments still have a very poor prognosis. Previous studies support a general anti-neoplastic effect of metformin, and a recent preclinical investigation has shown that metformin may control the expansion of *Dnmt3a* clonal hematopoiesis, which is known to precede MDS and AML.

**Patients/material and methods:**

In this study we investigated the effect of metformin and type 2 diabetes (T2D) on the risk of developing MDS or AML. T2D was defined based on hospital diagnosis codes and glucose-lowering drug prescriptions. The study was performed as a cohort study with follow-up from 1 January 2000 to 31 December 2017 using Danish national, population-based register data.

**Results and interpretation:**

In all, 6,031,132 persons contributed to the study of whom 302,403 had T2D, and 295,365 received metformin. Median follow-up time among individuals with T2D was more than 5 years, and among individuals without T2D more than 15 years. Our analyses revealed no association between T2D (hazard ratio [HR] 1.02 [95% confidence intervals (CI) 0.92–1.13]) or metformin use (HR 1.21 [95% CI 0.91–1.60]) and the risk of MDS or AML. However, when outcomes were studied separately, T2D was associated with an increased risk of MDS (HR 1.24 [95% CI 1.08–1.32]) but not with AML. Metformin use was not associated with MDS nor AML. Future studies should determine which patient groups may benefit from metformin to prevent MDS or AML development.

## Introduction

Myelodysplastic syndromes (MDS) and acute myeloid leukemia (AML) are myeloid neoplasms primarily affecting older individuals. For AML, treatment typically involves chemotherapy, and in high-risk cases, it may be combined with allogeneic bone marrow transplantation. For MDS, treatment usually consists only of allogeneic bone marrow stem cell transplantation. While the treatment landscape for MDS and AML has expanded over the last years, older or unfit patients, ineligible for curative treatment still have a very poor prognosis [1]. Alongside the development of novel, more effective therapies there is a need for strategies aimed at preventing the development of MDS and AML. With age, somatic mutations typical of MDS and AML accumulate in hematopoietic stem and progenitor cells. This phenomenon is known as clonal hematopoiesis of indeterminate potential (CHIP) and affects more than 10% of the healthy population above 70 years [2]. Individuals with CHIP, particularly those with concurrent cytopenia – referred to as clonal cytopenia of undetermined significance (CCUS) – are at an elevated risk of developing MDS and AML.

Currently, there are no well-established prophylactic treatment [3] but recent preclinical studies suggest that metformin may control the growth of myeloid clones carrying a *Ddmt3a* mutation, the most commonly mutated gene in CHIP and CCUS [4]. Metformin, a biguanide class anti-diabetic drug, is the first-choice anti-hyperglycemic treatment for patients with type 2 diabetes (T2D) [5]. Metformin has been used in diabetes treatment for over 60 years and numerous studies suggest a potential relevance in neoplastic diseases. Metformin modulates immune responses through multiple mechanisms, potentially enhancing its anti-neoplastic effects, particularly in cancers associated with immune dysfunction MDS [6, 7]. Several meta-analyses based on retrospective studies have demonstrated a decreased risk of cancer and improved cancer outcomes in patients with T2D treated with metformin compared to those treated with other anti-diabetic drugs [8, 9]. For hematological cancers, a lower risk of non-Hodgkin’s lymphomas and myeloproliferative neoplasms [10–13] as well as improved outcomes in patients with lymphomas and multiple myeloma have been reported [14–17]. A prospective cohort study found no significant association between the use of metformin versus other anti-diabetic drugs and the risk of developing leukemia in patients with T2D; however, the study did not distinguish between leukemia subtypes [18]. Conversely, T2D has been associated with a wide range of cancers and a poorer cancer prognosis, also in patients with hematological cancers [19, 20]. Studies investigating chronic and acute leukemias combined have identified an increased risk of leukemia in patients with T2D, with the majority of cases involving chronic leukemias [20, 21].

Currently, there is a lack of specific data on the association between metformin use for T2D and the development of MDS or AML. These patterns may differ from those observed in pooled analyses including chronic and lymphoid leukemias. In this study, we investigated the effect of metformin and T2D on the risk of developing MDS or AML using Danish national, population-based registers.

## Methods

### Data sources

The aim of this study was to estimate the impact of metformin and T2D on the risk of developing MDS or AML. Danish residents have a 10-digit civil registration number (CPR) used for all health care interactions, enabling individual-level linkage across various registers [22]. All persons above 18 years of age were eligible for follow-up through the Civil Registration System [23]. Information on International Classification of Diseases (ICD)-10 discharge diagnoses were available from The Danish National Patient Registry and Anatomical Therapeutic Chemical Classification (ATC) codes for prescriptions were available from The Danish National Prescription Registry [24, 25]. Data on MDS and AML diagnoses were obtained from The Danish National Acute Leukemia Registry, a clinical quality register containing information on all patients in Denmark diagnosed with AML since 2000 or with MDS since 2010 [26]. Reporting to the register is mandatory for all Danish physicians and the positive predictive values for the diagnosis is 99.6%. The way the cases are created in the clinical quality registers, with access to a wealth of electronic health records through many years from various sources ensures that for all practical purposes, incident cases are indeed incident cases. The criteria for defining T2D were consistent with the methodology previously described by our research group, that is either having a T2D diagnosis in the Danish National Patient Registry or receiving a prescription indicated by T2D [25, 27]. Metformin use was defined by prescriptions with ATC codes: A10BA02, A10BD02, A10BD03, A10BD05, A10BD07, A10BD08 A10BD10, A10BD11, A10BD13–A10BD18, A10BD20, A10BD22, A10BD23, A10BD25–A10BD27. Insulin and non-insulin diabetic treatment was defined by prescriptions with ATC codes A10A and A10B, respectively; the latter excluding metformin where appropriate.

### Study design and eligibility criteria

The cohort study aimed to elucidate the distinct effects of T2D and metformin treatment on the risk of MDS and AML, by only following individuals in one of three clinically meaningful states. In State 1, we included individuals diagnosed with T2D ≥1 year prior, who had been prescribed metformin within the past year; that is individuals supposedly in steady treatment for T2D with metformin. State 2 comprised individuals diagnosed with T2D ≥1 year prior, who had received ≥1 prescription for metformin previously, but not in the past year. Additionally, individuals in State 2 were currently being treated with at least one non-metformin anti-diabetic medication, that is people supposedly in steady treatment for T2D with one or more non-metformin anti-diabetics, presumably due to intolerance to metformin. To assess the impact of T2D itself, we included all individuals without any history of T2D or T2D medications in a control group designated as State 0.

Included individuals were followed for one of three endpoints from 1 January 2000 to 31 December 2017 provided they were alive and resident in Denmark and fulfilling the criteria for being in one of the three states. Thus, inclusion in the study is a time-dependent, dynamic process, where a person can potentially be followed in different states at different times, and do not necessarily have to be followed up in a contiguous fashion: they may be excluded from follow-up for example during longer stays abroad (not resident in Denmark) or during transitions from one state to another, while not fulfilling the criteria to be included in any of the states. All states are defined by the history of the patient, hence, by design, we do not at any time condition on the future and immortal time-bias cannot occur. The three endpoints considered were a diagnosis of MDS, AML, and either MDS or AML. We assumed current use of a given medication to have the same effect on risk of the outcome, regardless of how long the individual had been medicated, so that a given state would be the exposure. The underlying biological assumption being that medication would modify the risk of one or more steps in the process of turning a pre-malignant cell into a malignant cell.

### Statistical analysis

We performed descriptive statistics, presented as frequencies, percentages, and person-years. Hazard ratios (HRs) with corresponding 95% confidence intervals (CI) for outcomes were estimated using stratified Cox regression models, with birth year and sex as strata, and age as underlying timescale. To estimate the impact of metformin on the endpoints, we compared individuals in State 1 and State 2 and the impact of T2D was assessed comparing individuals in either State 1 or State 2 with individuals in State 0. We performed univariable and multivariable analyses adjusting for the Nordic Multimorbidity Index (NMI) score [28]. This choice of strata, timescale and adjustment was dictated by most risk factors (maleness, old age, smoking, certain chemicals, ionizing radiation, family history) for AML and MDS being rare or unavailable to us in the registers and in our computing environment, while on the other hand having easy access to sex, birth date (age), and the NMI mainly as a proxy for lifestyle [29]. CIs and *p*-values were based on Wald tests. Analyses were performed using SAS software, version 9.4 (SAS Institute, Cary, NC) on servers hosted by the Danish Health Data Authority.

### Ethics

This study was approved by SSI QC and Compliance (jr. no. 21/00805). All analyses were performed on pseudonymized data, and subgroup findings in less than five individuals were not reported, in accordance with requirements from the data steward (Danish Health Data Authority).

## Results

### Patient characteristics

A total of 6,031,132 individuals were enrolled in the study of whom 302,403 had or acquired T2D during follow-up (Table 1). The overall follow up-time for the cohort was 76,001,749 person years; 74,114,483 person-years were contributed by comparators and 1,887,266 person-years were contributed by persons with T2D. Median follow-up time among individuals with T2D was more than 5 years and among individuals without T2D more than 15 years (Table 1). Among individuals with T2D, 295,365 had received metformin, 83,859 had received insulin, and 156,347 had received non-metformin oral anti-diabetic drugs during follow-up. The 295,365 individuals treated with metformin collectively contributed 1,643,747 person-years, and 66,488 individuals treated with non-metformin anti-diabetic drugs (and previously treated with metformin) 243,520 persons years (Table 2).

**Table 1:** Characteristics during follow-up for persons with T2D and controls from the general population without T2D.

	T2D			No T2D		
	Number	Percent (%)	Person years	Number	Percent (%)	Person years
All	302.403	100.0	1.887.266	5.976.081	100.0	74.114.483
Sex						
Males	162.925	53.9	1.036.366	2.959.469	49.5	36.228.226
Females	139.478	46.1	850.900	3.016.612	50.5	37.886.257
Age, years						
18–39	26.375	8.7	64.268	3.441.747	57.6	27.481.012
40–49	43.560	14.4	152.063	2.198.950	36.8	13.788.512
50–59	94.041	31.1	365.653	2.084.156	34.9	12.669.170
60–69	137.315	45.4	592.369	1.649.044	27.6	10.193.807
70–79	124.673	41.2	493.180	1.181.270	19.8	6.318.193
80–89	58.121	19.2	197.526	630.347	10.5	3.047.774
≥ 90	9.932	3.3	22.208	185.204	3.1	616.015
Anti-diabetic treatment						
Metformin	295.365	97.7	1.643.747	.	.	.
Insulin	83.859	27.7	473.426	.	.	.
Non-insulin	299.170	98.9	1.753.200	.	.	.
NMI score						
0	186.299	61.6	559.679	5.527.250	92.5	48.898.802
2–4	190.818	63.1	372.291	4.576.442	76.6	13.205.396
> 3	228.303	75.5	955.297	2.965.154	49.6	12.010.285
Follow-up time, years						
0–4	302.403	100.0	1.123.367	5.976.081	100.0	26.166.332
5–9	160.959	53.2	538.288	4.702.277	78.7	21.412.653
10–14	64.955	21.5	197.081	3.882.154	65.0	17.607.229
≥ 15	19.368	6.4	28.530	3.185.701	53.3	8.928.268

T2D: type 2 diabetes.

**Table 2:** Characteristics during follow-up for persons with T2D treated with metformin or treated with non-metformin anti-diabetic drugs.

	Non-metformin			Metformin		
	Number	Percent (%)	Person years	Number	Percent (%)	Person years
All	66.488	100.0	243.520	295.365	100.0	1.643.747
Sex						
Males	35.276	53.1	124.486	159.543	54.0	911.880
Females	31.212	46.9	119.034	135.822	46.0	731.866
Age, years						
18–39	2.451	3.7	3.924	26.029	8.8	60.344
40–49	6.507	9.8	14.837	42.325	14.3	137.226
50–59	14.272	21.5	35.117	90.918	30.8	330.537
60–69	23.511	35.4	62.876	132.007	44.7	529.492
70–79	27.253	41.0	74.064	116.485	39.4	419.116
80–89	17.443	26.2	45.851	51.174	17.3	151.675
≥ 90	3.400	5.1	6.850	7.853	2.7	15.357
Anti-diabetic treatment						
Metformin	.	.	.	295.365	100.0	1.643.747
Insulin	40.108	60.3	167.054	74.880	25.4	306.372
Non-insulin	47.369	71.2	109.453	295.365	100.0	1.643.747
NMI score						
0	15.690	23.6	19.558	182.657	61.8	540.120
2–4	23.663	35.6	29.775	184.781	62.6	342.516
> 3	57.988	87.2	194.187	219.214	74.2	761.110
Follow-up time, years						
0–4	66.488	100.0	176.075	295.365	100.0	1.041.227
5–9	17.972	27.0	51.659	141.030	47.7	448.069
10–14	5.220	7.9	14.200	49.670	16.8	138.847
≥ 15	1.216	1.8	1.586	11.527	3.9	15.604

T2D: type 2 diabetes; NMI: Nordic Multimorbidity Index.

Individuals with T2D were most frequently contributed to the group aged between 60 and 69 years and tended to have an NMI score exceeding 3. In contrast, most persons without T2D contributed within the youngest age bracket (18–39 years) and had an NMI score of 0. Among persons treated with metformin, the most common age bracket was 60–69 years, whereas for persons in the non-metformin group it was 70–79 years. In the metformin group, 25.4% were concurrently treated with insulin and 51.0% with non-metformin oral anti-diabetic drugs. Among persons in the non-metformin group, 60.3% received insulin and 71.2% received non-metformin oral anti-diabetic drugs. Persons in the metformin group tended to have lower NMI scores than persons in the non-metformin group with 61.8% having a score of 0 compared with 23.6%. Sex was similarly distributed across all groups, with a balanced representation of both females and males.

### T2D and risk of MDS and AML

There were 2,246 cases of MDS and 4,563 cases of AML during follow-up, including 467 cases among individuals with T2D and 6,118 among T2D-naïve comparators. Table 3 presents HRs with corresponding 95% CIs and *p*-values for presence of T2D compared to its absence, as well as for metformin use compared to non-metformin anti-diabetics use. T2D was not associated with the risk of MDS or AML neither in the univariable analysis (HR 1.10 [95% CI 1.00–1.22], *p* = 0.060), or multivariable analysis adjusted for NMI score (HR 1.02; 95% CI 0.92–1.13). However, when MDS and AML were considered independently of one another, T2D was associated with an increased risk of MDS in both multivariable (HR 1.15 [95% CI 1.00–1.32, *p* = 0.048]) and univariable analysis (HR 1.24 [95% CI 1.08–1.42]). Meanwhile, no association was identified between T2D and risk of AML in univariable (HR 0.96 [95% CI 0.83–1.12]) or multivariable analysis (HR 0.89 [95% CI 0.76–1.03]).

**Table 3:** Hazard ratios with corresponding 95% confidence intervals and p-values of the associations between metformin and T2D with MDS and AML. All results are stratified by birth year and sex.

Outcome	Exposure	Adjusted for NMI	HR (95% CI)	*P*-value
AML or MDS	metformin	no	0.91 (0.69–1.20)	0.4971
AML or MDS	metformin	yes	1.21 (0.91–1.60)	0.1973
AML or MDS	T2D	no	1.10 (1.00–1.22)	0.0606
AML or MDS	T2D	yes	1.02 (0.92–1.13)	0.7127
MDS	metformin	no	0.86 (0.61–1.22)	0.4040
MDS	metformin	yes	1.22 (0.85–1.74)	0.2841
AML	metformin	no	0.96 (0.63–1.48)	0.8659
AML	metformin	yes	1.16 (0.75–1.79)	0.5183
MDS	T2D	no	1.24 (1.08–1.42)	0.0023
MDS	T2D	yes	1.15 (1.00–1.32)	0.0480
AML	T2D	no	0.96 (0.83–1.12)	0.6435
AML	T2D	yes	0.89 (0.76–1.03)	0.1183

T2D: type 2 diabetes; MDS: Myelodysplastic syndromes; AML: Acute myeloid leukemia; NMI: Nordic Multimorbidity Index; HR: Hazard raio; CI: confidence intervals.

### Metformin and risk of MDS and AML

There were 401 cases of MDS or AML in the metformin exposed cohort and 66 in the metformin unexposed cohort. Compared with use of other anti-diabetic drugs, metformin use was not associated with risk of MDS or AML neither in the univariable analysis (HR 0.91 [95% CI 0.69–1.20], Table 3) nor in the multivariable analysis additionally adjusted for NMI score (HR 1.21 [95% CI 0.91–1.60]). When MDS and AML were analyzed as separate outcomes, no association was found between metformin use and MDS or AML outcome whether in either univariable (MDS: HR 0.86 [95% CI 0.61–1.22]; AML: HR 0.96 [95% CI 0.63–1.48]) or multivariable analyses (MDS: HR 1.22 [95% CI 0.85–1.74]; AML: HR 1.16 [95% CI 0.75–1.79]).

## Discussion

This article presents findings from a study comparing the risk of developing MDS or AML in individuals with T2D treated with metformin and with other non-metformin anti-diabetic drugs, respectively. In addition, we assessed the risk of MDS and AML in individuals with T2D compared to individuals in the general population without T2D. Our study found an increased risk of MDS in individuals with T2D, but no association was observed between T2D and the risk of AML, or when MDS and AML were combined. Furthermore, there was no association between use of metformin or other anti-diabetic drugs and risk of MDS or AML. The lifetime-risk of MDS is approximately 0.77%, in Denmark [30, 31]. Thus, the relative increase in MDS risk identified (HR = 1.21) translates into an absolute increase in risk of at most 0.16%.

Several meta-analyses based on results from retrospective studies have suggested an association between T2D and an increased risk of cancers, including leukemia [19–21]. However, because the original studies included in these meta-analyses grouped chronic and acute leukemias together, as well as myeloid and lymphoid leukemias, the results have been difficult to interpret. To the best of our knowledge, this is the first study identifying an association between T2D and MDS. There is evidence to support that these conditions carry common genetic factors. *TET2* and *SF3B1* mutations have been found to present more frequently in the blood cells of MDS patients who also have diabetes [32]. A recent germline genetic study presented a set of 86 common susceptibility genes shared by T2D and AML that were related to metabolic cellular processes [33]. However, while T2D may correlate with an increased risk of many cancers, the impact of overweight and obesity on this relationship has not been fully established and could be a strong confounder or mediator of the association. *In vivo* and *in vitro* models have shown that AML cells may program bone marrow adipocytes to generate a pro-tumoral microenvironment promoting proliferation and survival of neoplastic cells [34]. Overweight and obesity have previously been associated with an increased risk of leukemia, however, similar to studies on T2D, these studies have not differentiated between the various types of leukemia [35]. In patients with MDS, obesity has been associated with shorter overall survival and an increased risk of transformation to AML [36]. Future prospective studies are needed to elucidate the impact of T2D, overweight, and related lifestyle factors such as diet and exercise on the development of MDS and AML.

Since metformin is the first-choice treatment for patients with T2D, studying its effects in a non-randomized cohort naturally introduces biases. Various factors may lead to the decision not to initiate metformin in a person with T2D, such as effective management through dietary intervention, patient preference, impaired renal function, or severe comorbidities that make insulin treatment more appropriate. Comparing T2D patients treated with metformin to metformin-naïve T2D patients could result in significant biases due to differences in baseline cancer risk factors such as comorbidities, diet, and lifestyle. To mitigate this bias, we compared individuals currently receiving metformin with individuals who had previously used it but were no longer on the medication. While there may be various reasons for discontinuing metformin, we hypothesized that side effects would be the most common reason, thereby limiting the differences in characteristics across patient groups. However, as evident from the distribution of NMI scores and age brackets, the metformin unexposed group was more comorbid and older compared with the metformin group. By using age as the underlying time scale (and thereby adjusting for age as detailed as possible) and adjusting for NMI scores we sought to enhance the group comparability. Moreover, patients who use additional glucose-lowering medications alongside metformin may differ in key characteristics from those on metformin monotherapy, introducing potential indication bias. However, restricting the analysis to metformin monotherapy users would yield a small cohort in a real-world setting, leading to imprecise estimates. Therefore, we have prioritized studying a more heterogeneous population to enhance the likelihood of detecting a signal if present. Finally, as this is a population-based study, we can only infer potential associations and not causation.

Although our study did not find any association between the use of metformin among individuals with T2D and the risk of AML or MDS previous studies assessing the risk of various hematological malignancies with metformin use have reported a decreased risk with effect sizes ranging from HR/odds ratio 0.33 to 0.85 [11–13, 18]. The mechanism of action of metformin in T2D is only partly understood; however, its impact on energy metabolism through inhibition of hepatic glucose production is considered a key mechanism [37]. Even less is known about the mechanisms of metformin in cancer, but both direct and indirect effects on cancer cells have been suggested, including a lowering of insulin levels which may suppress cancer cell proliferation [38, 39]. Preclinical studies suggest metformin’s function is related to specific mutations in genes associated with myeloid cancer [40]. Given that MDS and AML are highly heterogeneous diseases, it is possible that certain subtypes of myeloid cancer may be targetable through metformin prevention strategies. The emerging potential of metformin as a cancer preventive agent prompts investigation into its optimal repurposing strategies for target populations. A potential approach for preventing hematological cancers in individuals with precursor conditions involves identifying and targeting high-risk groups or individuals with specific mutations that are presumed to be responsive to metformin. Given the well-known association between hyperglycemia, insulin resistance and cancer, T2D may not be the best model for investigating a potential role of metformin as a leukemia prophylactic drug. To that end, we are currently conducting a pilot clinical trial to investigate safety, feasibility and activity of metformin treatment in patients with CCUS or lower-risk MDS who do not have T2D (NCT04741945). However, in the ongoing trial, the presence of specific mutations potentially responsive to metformin based on findings in recent preclinical studies is not an inclusion criterion [4].

## Conclusions

This study is the first to identify an increased risk of MDS in individuals with T2D. We found no association between metformin use and the risk of developing MDS or AML. Previous studies suggest that metformin has broad anti-neoplastic effects and preclinical studies have shown inhibitory effects of metformin on specific pre-leukemic myeloid clones. Future prospective studies are necessary to determine if subgroups of individuals with low-risk myeloid cancers or precursor conditions may benefit from metformin to prevent development of higher-risk MDS or AML.

## Data Availability

Data will be made available upon reasonable request and in accordance with Danish legislation.
